# Illuminating Airway Nerve Structure and Function in Chronic Cough

**DOI:** 10.1007/s00408-023-00659-x

**Published:** 2023-11-21

**Authors:** James Kornfield, Ubaldo De La Torre, Emily Mize, Matthew G. Drake

**Affiliations:** OHSU Division of Pulmonary, Allergy, and Critical Care Medicine, Oregon Health & Science University, 3181 SW Sam Jackson Park Rd, Mail Code UHN67, Portland, OR 97239 USA

**Keywords:** Chronic cough, Sensory nerve, Parasympathetic nerve, Asthma, Confocal microscopy, Optogenetics

## Abstract

Airway nerves regulate vital airway functions including bronchoconstriction, cough, and control of respiration. Dysregulation of airway nerves underlies the development and manifestations of airway diseases such as chronic cough, where sensitization of neural pathways leads to excessive cough triggering. Nerves are heterogeneous in both expression and function. Recent advances in confocal imaging and in targeted genetic manipulation of airway nerves have expanded our ability to visualize neural organization, study neuro-immune interactions, and selectively modulate nerve activation. As a result, we have an unprecedented ability to quantitatively assess neural remodeling and its role in the development of airway disease. This review highlights our existing understanding of neural heterogeneity and how advances in methodology have illuminated airway nerve morphology and function in health and disease.

## Introduction

Airway nerves serve critical functions in the upper and lower respiratory tract including regulation of breathing and control of bronchoconstriction and cough. Both afferent and efferent fibers contribute to these functions and represent numerous neuronal subsets, each contributing discrete input to the regulation of airway functions. Numerous methods for classifying airway nerve subtypes have been proposed based on neuronal structure, expression, or function, yet no single classification scheme fully encapsulates airway neuronal diversity. Single-cell RNA sequencing techniques have highlighted this diversity by identifying at least 18 unique transcriptomic subtypes of sensory nerves alone [1]. Nerve subtypes frequently exhibit overlapping expression of receptors, neurotransmitters, and neuropeptides, further underscoring the challenge of creating a unifying classification scheme [2]. However, recent technological innovations in confocal microscopy and advances in genetic manipulation have provided new opportunities for studying nerve structure, expression, and function for both common and rare neuronal subtypes in the lungs. Here, we describe recent insights derived from studies using novel methods, with a focus on airway neural organization in healthy lungs and the role of neural remodeling in the pathogenesis of chronic cough.

## Neurologic Origins of Chronic Cough

Cough is a protective response that clears pathogens and mucus from airways and is regulated by airway sensory nerves [3]. To produce an effective cough, sensory input must be integrated in the brainstem to evoke responses in skeletal nerves and efferent airway nerves produce a deep inspiration followed by forced exhalation against a closed glottis [4]. The necessity of effective coughing to lung health is underscored by the increased frequency of pneumonia in conditions where cough is impaired [5].

Unlike protective cough, chronic cough represents a pathologic state that no longer serves a physiologic role. Chronic cough is a central feature that develops in a myriad of lung diseases [3]. That chronic cough is shared by diseases with disparate pathologies, such as asthma (an inflammatory airway disease) and idiopathic pulmonary fibrosis (an alveolar fibrosing disease), highlights the significant role that dysregulated airway nerves play in the clinical manifestations of lung disease. Patients with chronic cough frequently report an urge to cough coupled with an irritation or “itch” sensation in the throat and a heightened sensitivity to environmental triggers such as cold air or perfumes. These symptoms, which have been termed “cough hypersensitivity,” develop due to sensitization of neuronal pathways that govern cough and contribute to excessive cough triggering [6].

Cough challenge studies suggest that neuronal sensitization is a heterogeneous process that results in distinct neurophenotypes, as reflected by differing cough responses to inhaled stimuli between airway diseases [7]. For example, cough sensitivity to inhaled capsaicin (an agonist of neuronal transient receptor potential (TRP) V1 was similar between patients with chronic obstructive pulmonary disease and chronic idiopathic cough, while sensitivity to inhaled prostaglandin E2 was significantly different. These unique cough neurophenotypes are predicted to result from different mediators and mechanisms driving development of each disease.

## Organization of Airway Innervation in Healthy Lungs

### Sensory Afferent Innervation of the Larynx and Lower Airway

Sensory innervation of the lower airways, extending from the larynx proximally to the distal terminal bronchioles, is provided primarily by fibers contained with the vagus nerves, with minor contributions provided by sensory neurons from the thoracic dorsal root ganglia [2, 8, 9]. Vagal sensory nerve cell bodies are contained within the jugular (superior) and the nodose (inferior) ganglia, collectively termed the vagal ganglia, located at the base of the skull [10]. These ganglia have distinct embryological origins and targets. Jugular nerves are derived from neural crest cells and primarily innervate the trachea and large airways, whereas nodose nerves are derived from epibranchial placodes and provide innervation to distal airways and lungs [11]. Sensory axons terminate within all major compartments of the airways including the epithelium, subepithelium, and smooth muscle, while also providing discrete innervation to airway mucus glands, autonomic ganglia, alveolar capillary beds, and other airway structures [12–14].

Sensory nerves can be broadly classified as mechanoreceptors or chemoreceptors based on their responsiveness to mechanical or chemical stimuli. Mechanoreceptors are typically larger myelinated fibers that are highly sensitive to touch, whereas chemoreceptors (also termed nociceptors or C fibers) are typically small-diameter, unmyelinated fibers that express a wide array of receptors and ion channels capable of detecting inhaled and endogenous noxious compounds, and changes in pH, temperature, and osmolarity [10, 15–18]. Receptors with specific relevance to cough (both to cough triggering and in the pathogenesis and potential treatment of chronic cough) include P2X3 purinergic receptors, voltage-gated sodium channels (NaV), bradykinin receptors, and TRP channels (discussed below) [19, 20]. Mechanoreceptors can be further subclassified as slowly adapting and rapidly adapting based on their speed of adaptation to sustained stimuli and their ability to modulate respiratory patterns and cough responses. Sensory nerve input is transmitted to the paratrigeminal nucleus (jugular) and nucleus of the solitary tract (nodose) within the brainstem [21–23]. Input from both mechanoreceptors and nociceptors can trigger cough, during which the respiratory pattern generator of the brainstem switches from a rhythmic breathing pattern to a cough pattern. Sensory input is also transmitted to efferent airway nerves to induce reflex bronchoconstriction and to higher-order cortical neurons where conscious perception of cough and cough suppression centers may modulate coughing.

### Efferent Innervation of the Lower Airways

The primary efferent innervation of the airways is provided by cholinergic parasympathetic nerves, which provide the dominant control of bronchoconstriction [24]. Preganglionic parasympathetic neurons originate in the dorsal motor nucleus and nucleus ambiguus in the brainstem, travel within the vagus nerves (alongside sensory afferents), and synapse on postganglionic nerves contained in airway ganglia seated in the walls of the trachea and extrapulmonary bronchi [25–28]. Post-ganglionic processes branch extensively throughout the tracheobronchial tree to terminate on submucosal glands [12, 29], blood vessels [13], and most prominently, airway smooth muscle [30], where they release acetylcholine to induce smooth muscle contraction via M3 muscarinic receptor activation. Acetylcholine also binds prejunctional M2 muscarinic receptors, which provides an inhibitory feedback mechanism limiting further acetylcholine release [31–36]. Parasympathetic acetylcholine release is triggered by input from the cortex and by direct stimulation from sensory nerves in the brainstem [14, 37]. Bronchoconstriction resulting from sensory nerve-mediated parasympathetic nerve activation is termed “reflex bronchoconstriction.” Reflex bronchoconstriction has been demonstrated in both humans and animals, and in response to a variety of sensory nerve stimuli including histamine [37], methacholine [38], allergen [39], cold air [40], and exercise [41].

In addition to parasympathetic nerves, sympathetic and non-adrenergic non-cholinergic nerves provide additional efferent innervation of the lower airways [42]. In humans, sympathetic fibers principally innervate airway vasculature, with essentially no direct input to airway smooth muscle (in contrast to the sympathetic innervation of smooth muscle in mice) [43]. In contrast, non-adrenergic non-cholinergic (NANC) nerves induce airway smooth muscle relaxation through release of nitric oxide (NO) and vaso-intestinal peptide (VIP) [44–47].

## Advanced Methods for Studying Airway Innervation

### Confocal Microscopy Illuminates 3-Dimensional Complexity of Sensory Innervation

**Fig. 1:**
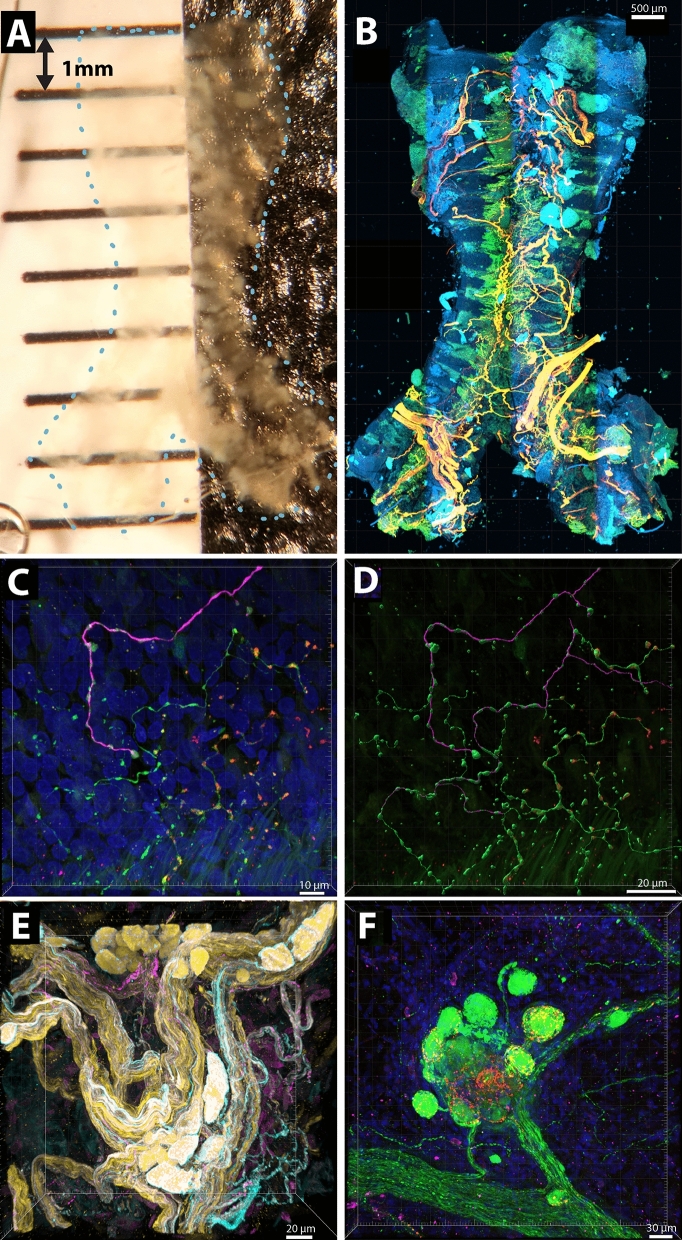
Tissue optical clearing and high-resolution confocal microscopy enable quantitative modeling of airway nerve morphology. **a** Mouse trachea immunostained and cleared using an optical clearing reagent to render tissue transparent. **b** Immunolabeling of transparent airway tissues followed by confocal imaging provides detailed visualization of airway nerves. Orange: pan-neuronal marker AbPGP9.5; Green: channelrhodopsin-CH2; Blue: nuclear stain DAPI. Scale bar: 500 µm. **c** Epithelial sensory nerves in a human bronchiole immunostained for PGP9.5 (green), NFHC (magenta), and neuropeptide substance P (red). Scale bar: 10 µm **d** 3D nerve model based on PGP9.5-positive voxels in C using Imaris software; Scale bar: 20 µm. **e** Parasympathetic ganglion in optogenetic mouse trachea immunostained for PGP9.5 (yellow), channelrhodopsin-CH2 (turquoise) and neuropeptide substance P (magenta). Scale bar: 20 µm. **f** Parasympathetic ganglion in human lung immunostained for PGP9.5 (green), substance P (red), and TRPV1 (magenta). Scale bar: 30 µm. *PGP9.5* protein gene product 9.5, *CH2* channelrhodopsin-2 expressed on parasympathetic ganglia, *TRPV1* transient receptor potential vanilloid subtype 1, *NFHC* neurofilament heavy chain

Airway sensory nerves form complex, 3-dimensional structures that can span hundreds of histologic tissue sections. This complexity has made studying the morphology of airway nerves in individual tissue sections challenging. Heterogenous expression of receptors and neuropeptides by airway sensory nerves has further complicated quantitative assessments of nerve morphology [48]. However, advances in confocal imaging and immunohistochemistry have bridged this technological gap by capturing high-resolution, 3-dimensional image Z-stacks of airway structures using whole-mount tissues that do not require tissue sectioning. When paired with tissue optical clearing, where airway specimens or whole lungs are rendered transparent by an optical clearing reagent, image Z-stacks can extend through entire organs, limited only by the optical constraints of the confocal objectives (Fig. 1a, b) [49, 50].

Confocal techniques have illuminated a remarkable degree of three-dimensional neural complexity in both human airways [51–53] and animal models, including rat [54], pig [55], rabbit [56], guinea pig [57], and mouse [49, 50]. Nerves are interposed within and around virtually all airway structures and in all tissue layers (e.g., epithelium, subepithelium, smooth muscle, etc.) [58]. Epithelial sensory nerves, for example, extend from subepithelial roots to form a lattice of branching nerve terminals among airway epithelial cells in close juxtaposition to airways, where they detect impacting particles and noxious compounds (Fig. 1c, d). Sensory nerve density (i.e., total nerve length) and complexity (i.e., nerve branching) vary by airway location and are tissue-compartment specific, with both density and complexity decreasing from proximal trachea to bronchi, and from the dorsal to ventral aspect of the airway [49, 50]. Epithelial nerve complexity is also greatest at airway branch points, where inhaled particles are most likely to impact, and surrounding airway parasympathetic ganglia embedded within the airway wall (Fig. 1e, f) and among specialized cells embedded within the epithelial layer termed neuroepithelial bodies (NEBs) [59]. Collectively, NEBs and the nerve axons surrounding them are termed pulmonary neuroendocrine cells (PNECs), and are composed of multiple TRPV1 and substance P-expressing nociceptive C fibers, and cholinergic neurons [60], suggesting that these cells functionally serve mechanosensory, chemosensory, and cholinergic roles. PNECs are sparsely and randomly distributed throughout human airways [61].

### Fluorescence Labeling Highlights Axonal Organization

As nerve axons travel deeper into tissues (i.e., from epithelium to subepithelium and smooth muscle), they frequently join larger nerve bundles consisting of a mix of sensory and efferent fibers. Techniques have been developed to trace nerve axons to their termination, including the application of lipophilic dyes (e.g., carbocyanine dye DiI) [62], horseradish peroxidase [63], and wheat germ agglutin [64]; methods that involve uptake and transport of tracers along axons to enable visualization of nerve course and synaptic organization. Su et al. recently combined retrograde tracing methods, immunohistochemistry, and confocal imaging to show that central projections of TRPV1 and substance P-positive neurons within vagal ganglia have both shared and distinct synaptic targets in airways, including within airway smooth muscles, along lymphatic, and surrounding alveoli [65]. Confocal immunostaining has also demonstrated patterns of overlapping expression patterns on sensory neurons. For example, on nociceptors, the most commonly expressed receptors and peptides included Trpv1 (78%), Piezo1 (74%), Piezo2 (69%), and substance P (57%), followed by Calb1 (45%), Trpa1 (48%), and VIP (24%) . Receptors and peptides frequently colocalize (i.e., dual TRPV1 + and substance P + sensory neurons), with each unique combination of co-expression representing a small portion of total nerves overall [57, 66].

Neuronal organization and expression have been defined using Cre-lox-based genetic reporter mice coupled with fluorescent proteins. Using Pirt+, 5HT3+, substance P+, and TRPV1 + reporters, this method demonstrated relative contributions of each neuronal subtype to the innervation of airway targets [67]. Piezo2 reporters have also been used to elucidate their functional roles in detecting pulmonary stretch [68].

Multi-color nerve labeling is an alternative method for axonal tracing. Unlike retrograde tracers or Cre reporter mice, which label nerves originating from a common site (i.e., airway lumen) or expressing a specific promoter with a single color, multi-color nerve labeling provides a distinct fluorescent color for each nerve process, enabling distinction of individual axons in close proximity and tracing of individual nerves to their target of innervation [69]. Multi-color labeling has been used to study neurons in the brain (e.g., Brainbow mice [70]), where fluorophore expression was driven by the Thy1 promoter. Peripheral nerves had not been studieds in this manner due to the absence of Thy1 promoter expression by peripheral neurons. We used a modified technique involving simultaneous injection of three neurotrophic adeno-associated virus (AAV) vectors tagged with a distinct fluorophore to produce a spectrum of colors in airway neurons [69]. Random viral transduction within each neuron produces different ratios of fluorophore expression to enable distinction and tracing of individual nerve axons. When paired with conventional immunohistochemistry, the morphology of specific nerves, such substance P, neuronal NOS-, and TH-expressing neurons, can be traced to their termination.

### Confocal Studies of Axonal Development During Embryogenesis

During embryogenesis, airway neuronal outgrowth is closely associated with airway elongation and airway smooth muscle proliferation [71]. These primitive airway tubules are coated by a dense neural plexus overlying smooth muscle, which by the canicular phase, forms two distinct bronchial trunks giving rise to varicosed fibers and discrete airway ganglia [72]. Similar patterns of neuronal elongation and branching have been demonstrated in the pig, rabbit, and mouse fetal lung, where neural tissue is a dominant feature of the developing lung [61, 72–74].

Vagal sensory input supplies an abundance of fibers to cholinergic airway ganglia precursors as well. These ganglia, which originate from neuroblasts along the wall of the epithelial tubules during the pseudoglandular stage, coalesce and become increasingly enveloped by glial fibrillary acid-positive sheaths. Ganglionic neurons transition to a cholinergic phenotype from the canalicular stage onward, further increasing in size during the saccular phase and during early post-natal development [73].

### Transgenic Models for Testing Airway Nerve Function

Testing the function of activated airway nerves has historically required electrical stimulation, either via electrodes attached to nerve bundles in vivo (e.g., the vagus nerve trunks) or by applying electrical currents across isolated airway segments ex vivo [75], or through the application of pharmacologic agonists. While these techniques contributed significantly to our understanding of neural control of airway function, their readouts were limited by a lack of selectivity for neuronal subtypes. Recent applications of transgenic and Cre-recombinase-based methods, such as optogenetics and in vivo calcium fluorescence, have significantly advanced our ability to manipulate and measure the function of neuronal subtypes.

Optogenetics involves genetic insertion of photosensitive ion channels into specific neuronal subpopulations, enabling targeted nerve activation or inhibition using light [76]. While discovery of opsin-based channels is now over 20 years ago, genetic insertion techniques and channel options continue to expand, providing increasingly selective control of nerve function. Our lab has applied this approach to provoke or inhibit nerve-mediated bronchoconstriction in vivo by inserting nerve-activating channelrhodopsin and nerve-inhibiting halorhodopsin channels into efferent choline-acetyltransferase-expressing cholinergic parasympathetic nerves and into advillin- and tac1-expressing sensory nerves [77, 78]. Similarly, optogenetic activation of TRPV1- and S1PR3-positive sensory nerves stimulated bronchoconstriction in allergen-sensitized mice [79]. Activation of P2RY1-expressing sensory neurons triggered a series of reflexes designed to prevent aspiration, including pharyngeal swallowing, apnea, and vocal fold adduction [80, 81], while Piezo2-expressing sensory neurons, which often also co-express P2RY1, produced sustained apnea upon optogenetic light stimulation without pharyngeal and vocal cord reflexes, suggesting that Piezo2 neurons provide mechanosensory feedback of lung stretch during physiologic respiration [68].

Cre-lox recombination has also been used to insert calcium-sensitive fluorophores into neurons to study nerve activation in vivo at a single-cell resolutionwith two-photon microscopy [82]. In this study, Pirt-cre mice in which cre recombinase is expressed in all vagal neurons were crossed with R26‐GCaMP6s to create a strain that expresses a calcium-sensitive fluorophore in vagal sensory neurons. The effects of the lipid agonist sphingosine‐1‐phosphate (S1P), which is elevated in asthma, were then tested in vivo. Approximately 80% of vagal sensory neurons responded to SP1 via S1PR3 receptors, suggesting that elevated S1P levels in inflammatory conditions like asthma contribute to increased neuronal activation in diseased lungs.

## Neuronal Remodeling and Neuro-immune Interactions—Implications for Chronic Cough Pathogenesis

### Neural Sensitization Contributes to Excessive Cough

Several mechanisms have been identified, which may contribute to neuronal sensitization in chronic cough, including increased nociceptor sensitivity, de novo expression of nociceptors and neuropeptides by sensory neurons, increased airway epithelial nerve density, and increased release of endogenous cough-triggering molecules in airways [83]. While most of these mechanisms are derived from animal models, we recently demonstrated in bronchoscopic human airway samples using tissue optical clearing and confocal microscopy that airway epithelial sensory nerve density is doubled in patients with chronic cough compared to healthy airways [53]. In some samples, sensory neuropeptide substance P was also increased although not uniformly in the chronic cough cohort, in line with cough challenge studies suggesting that heterogeneous neuronal remodeling events may underlie the development of clinical symptoms [7].

Substance P augments cough responses by lowering neuronal activation thresholds [84, 85]. However, its role in chronic cough has been in doubt since an initial clinical trial of a substance P receptor (neurokinin 1 and 2) antagonist failed to reduce cough frequency [86]. Since that study, a second family of substance P receptors (mas-related g-protein coupled receptors, Mrgprs) has been discovered, which has been linked to the generation of itch; a sensory nerve-mediated process in skin with many similarities to cough [87]. This pathway, coupled with the identification of distinct cough neurophenotypes and our finding that substance P is increased in human airways, suggest that neurokinins require a fresh examination as a therapeutic target. Indeed, two recent studies of the NK-1 receptor antagonist, aprepitant, reported a decrease in cough frequency compared to placebo in patients with lung cancer and chronic cough [88, 89]. A second NK-1 receptor antagonist is also under clinical investigation [90]. Whether this approach will be broadly applicable across diseases or more targeted for specific populations awaits further study.

Several sensory receptors have also been implicated in chronic cough generation. Foremost are P2X3 purinoreceptors, which are expressed by approximately 1/3 of nodose sensory neurons [91, 92] and are activated by ATP, an endogenous mediator released during times of cell stress. In diseased lungs, cough responses to inhaled ATP are increased [93–95]. Moreover, P2X3 antagonists in phase 2 and 3 trials have demonstrated reductions in cough frequency [96–102]. If approved for clinical use, P2X3 receptor antagonists would represent the first-targeted therapy approved for chronic cough.

How P2X3 signaling is modulated in disease is an area of active interest. Increased extracellular ATP has been reported in asthma and chronic obstructive pulmonary disease, which may contribute to P2X3-mediated cough [103, 104]. However, this finding would not explain the increased sensitivity to inhaled ATP in chronic cough [95]. Rather, we hypothesized that neuronal P2X3 expression is increased by airway inflammation. To test this postulate, we quantified airway neuronal P2X3 expression in a mouse model of eosinophilic asthma (a disease frequently associated with chronic cough). In these mice, neuronal P2X3 expression was significantly increased compared control animals, suggesting modulation of P2X3 expression may underlie development of ATP sensitivity in some cases [105]. Modulation of endogenous ATP release may also occur, either through alterations in the number or function of ATP-releasing Pannexin-1 channels on structural and inflammatory cells directly, or indirectly via modulation of pathways that regulate Pannexin-1 function, as suggested by Bonvini et al. who reported that TRPV4 agonist stimulated Pannexin-1 ATP release to evoke cough [106].

TRP channels, including TRPV4 as well as TRPV1, TRPA1, and others, are a family of transmembrane proteins that detect a wide array of cough-provoking irritants [107–111]. Cough studies in guinea pigs have revealed a positive correlation between cough frequency and TRPA1 and TRPV1 expression [112], and increased TRPV1 channel expression has been demonstrated in airways of humans with chronic cough [113, 114]. In patients with asthma and in those with chronic obstructive pulmonary disease (both conditions associated with chronic cough), TRPV1 expression and cough responses to TRPV1 agonists are increased [7]. In animal models, mechanoreceptors express TRPV1 de novo after allergen exposure and virus infection [115–117], possibly due to induction of the neurotrophin brain-derived neurotrophic factor (BDNF) [115].

Despite a clear role for TRP channels in triggering cough and TRP channel antagonists’ efficacy in blocking evoked cough responses, multiple TRP antagonists have failed to reduce cough frequency in chronic cough clinical trials [118–120]. This apparent discrepancy highlights the challenge in developing anti-tussives that block pathologic cough while preserving protective cough.

### Neuro-immune Interactions Result in Dysregulated Airway Function

Chronic cough is a common feature in over 100 distinct diseases, many of which are characterized by the influx of inflammatory cells into airways. As an example, asthma is an inflammatory airway disease characterized by excessive bronchoconstriction and in many cases, chronic cough, with increased sensitivity to inhaled irritants [121]. In asthma, airway eosinophils, which are abundant in a majority of patients, migrate to nerves due to neuronal release of the eosinophil chemoattractant eotaxin [122–124]. Eosinophil’s interactions with nerves have profound effects on both afferent and efferent pathways. Airway eosinophils were associated with increased epithelial sensory nerve density in bronchoscopic airway samples from humans with asthma and were demonstrated to mediate sensory hyperinnervation in mice (quantified using confocal microscopy) [52]. In mice, increased sensory nerve density develops after chronic allergen-induced eosinophilia (i.e., house dust mite allergen exposure for 8 weeks) and in offspring exposed to maternal asthma in utero, suggesting hyperinnervation develops due to prenatal programming, predisposing an individual to lung disease later in life [125, 126]. These morphologic changes in airway nerves, which are akin to those seen in idiopathic chronic cough patients [53], increase bronchoconstriction evoked by sensory nerve activation and were associated with increased sensitivity to environmental irritants [52]. Eosinophils also exacerbate efferent parasympathetic nerve control of bronchoconstriction [122, 127, 128]. Thus, both bronchoconstriction and cough, which are cardinal signs of asthma, result from inflammatory cell effects on each limb of airway innervation.

Eosinophil proximity to nerves is critical to the development of nerve dysfunction. To study the effects of eosinophil proximity on nerve structure and function, we paired in vivo measurements of bronchoconstriction using optogenetic mice with confocal imaging to quantify spatial interactions between leukocytes and their effects on neuronal subtypes. We demonstrated that the density of tissue eosinophils is significantly increased around airway nerves, which correlates with increased neuronally-mediated bronchoconstriction [77, 78]. The combined effects of eosinophil interactions with nerves, coupled with pre-existing airway hyperinnervation, were profound, resulting in fatal bronchoconstriction in a mouse model of asthma [125]. Thus, structural remodeling coupled with physical interactions with eosinophils severely dysregulates neural control of airway tone.

## Conclusions

Airway nerves are heterogeneous, with overlapping patterns for receptors and protein expression that define their functional role in regulating cough, bronchoconstriction, respiration, and other functions. Neuronal remodeling underlies the development of airway disorders, including most prominently, chronic cough. Advances in confocal imaging and genetic methods have expanded our understanding of the function and morphology of neuronal subtypes, while enabling quantitative analyses of neuronal remodeling and neuro-immune interactions. These results offer new insights into mechanisms of disease pathogenesis and potential treatment targets, for which targeted therapies in chronic cough are urgently needed.
